# Introductory Lecture

**Published:** 1872-01

**Authors:** Walter Hay

**Affiliations:** Asst. to the Prof. Prin. and Prac. Med., Rush Medical College


*Anæsthesia, Hyperæsthesia.*—Introductory Lecture by WALTER HAY, M.D., Asst. to the Prof. Prin. and Prac. Med., Rush Medical College.

*Gentlemen:* In appearing before you at the courteous invitation of the accomplished gentleman who so ably fills this chair, and by the permission of the Faculty of Rush Medical College, I desire to acknowledge, in advance, my appreciation of the very great honor which they have done me in assigning to me the very pleasing duty of instructing you in the outlines of the diseases of the nervous system. I say the outlines of the subject, for of a subject so vast, so important, so intricate, and so intimately correlated with every pathological problem which can ever be presented to you in your future professional career, it would be impossible for any one, in a single course of lectures, to do more than to designate the outlines, to indicate the prominent landmarks, and to lay down the principles which should constitute the bases of your observations and studies of details hereafter.

In claiming for the pathology of the nervous system the most important place in the whole cycle of the physician's practical studies, I may cite this fact in evidence, that just in proportion to its development and differentiation, is the organism elevated in the scale of creation; and just in proportion to its restriction is the organism degraded. And this is true of the whole of organized nature in general, as of every individual in detail.

Beginning with the lowest member of the animal series and noting carefully the superaddition of one function after another, to each organism, as we ascend the scale of creation, as their relations become more and more complicated, we shall perceive that coincidentally with every additional function, an addition has been made to the nervous apparatus for the domination and control of that function. So also in the reverse order, in descending from the highest to those lower in the scale of organized beings, as function becomes more and more restricted, as the relations of the being become more and more contracted, we shall observe the abstraction of portion after portion, ganglion after ganglion of the nervous system.

Again, turning for our illustrations to the field of pathology, we see the same parallelism between abolition of function and loss of structure as has been already adverted to in the physiological series; loss of voluntary motion, with lesion of the antero-lateral columns of the spinal cord, loss of sensation, with disorder of the posterior gray substance of the same.

Magendie, one of the fathers of modern physiology, called these the functions of relation, so especially is their office an internuncial one—that of maintaining our relations with all that is outside of ourselves, and the correlations of every internal organ with all the others, by which means structural integrity, and, necessarily, functional equilibrium, is maintained.

But there is one prerequisite essential to a correct appreciation of the morbid conditions of the nervous system which we are about to discuss—if such distinctions were admissible, I might say more essential than to that of any other field of research—and this is, a knowledge, comprehensive and extensive, of the anatomy and physiology of the nervous system. Whatever you may be inclined to neglect, in the course of your studies, cultivate these with all the ardor in your power. For this you have facilities which have been enjoyed by few; facilities by means of which you are instructed directly in the operations of physiological laws, which have become known to your predecessors only through long years of patient toil and laborious induction.

There is probably, at this time, no institution in the country where the science of physiology is taught more thoroughly, more practically, and more efficiently than in this; and very few, if any, where the advantages for the prosecution of this all-important study even approximate to those which are afforded to you here. Not being a member of its Faculty, nor even one of its graduates, I feel the more freedom in expressing an honest opinion upon this head.

Understanding then, thoroughly, the mechanism of this organism, and the laws governing its operations, structure and function, anatomy and physiology, you will then be prepared to appreciate the perturbations which they may sustain under the operation of certain determinate causes, and, conversely, the causes to which these disturbances are due. But at this point it is necessary for me to warn you of the importance of bearing this fundamental truth in your minds, without a knowledge of which your ideas of nervous pathology will be mere speculation, and your therapeutics empiricism. The truth which I wish to impress on you is this, that for every variation of functional energy there is a change of structure coincident. All diseases of the nervous system, as indeed all others, necessarily involve organic changes.

Now with regard to the causes which determine disturbances in the nervous system, it will be necessary for you to consider a very wide range of agencies; for the day has gone by when any theory of specific causation can be regarded as sufficiently comprehensive to satisfy the inquiring mind of the rational physician. It will not be sufficient for you to determine that the convulsions of one patient were caused by dysmenorrhea, because the insanity of another may have the same antecedent. This sort of ætiology will do well enough to accompany the therapeutics which prescribes "castor oil as good for the diarrhœa," and opium as good for metritis, and, combined with a little more knowledge of the same sort, goes to constitute a popular "ism," termed empiricism, with which you of course have nothing to do.

You must study the nature and influence of concurrent causes. Study the relations, to your patient, of locality, climate, hereditary proclivities, diathesis, sex, age, social and sanitary condition and surroundings, occupation, personal habits and characteristics. For upon this head it will be necessary for you to remember, what has been already said, that, as the nervous system is the medium by which we are brought into relation with everything, objective and subjective, with all that is without and all that is within ourselves, by its disturbance these relations must necessarily be perverted; and conversely with their perversion the nervous system will be disturbed.

In a word, just as the engineer fixes forever the exact locality, to a hairsbreadth, of the point from which his observations are made, by measuring the bearings and distances of many surrounding, permanent objects from it, and, conversely, of it from them, so do you measure the bearings and distances, so to speak, of all these concurrent causes upon the case before you, and you will thus truly make a diagnosis (which is a knowing of something through some other things,) and be enabled thereby to arrive at a true therapeusis.

For example, when a patient is suffering from pain, it will not be sufficient for you to give him morphine, because morphine will allay pain; but, having first determined the cause of the pain, or, at least, its nature, and the absence of any contra-indications, then remember what Dr. Chambers has so eloquently said in this regard: "Pain is the cry of a starved nerve for food." Now the blood is the "pabulum vitæ," and hence the pabulum nervorum. Morphine, by paralyzing the vaso-motor nerves, permits the relaxation of the middle or muscular coat of the arteries, and the consequent distention of the arterial calibre, thereby diminishing the rapidity of the blood current and the waste of tissue, allowing thereby more time for repair. The integrity of the nerve tissue is thus protected from the disintegrating chemico-vital agencies, and it is therefore enabled to convey a normal and not a morbid and exaggerated sensation to the sensorium.

In describing to you the diseases of the nervous system as they are at present understood, I shall do so in the reverse order to that in which they are usually arranged. That is, instead of beginning with the diseases of the eucephalon, and descending to those of the spinal cord, which is the course usually pursued, and which has the sanction of time and prescript, and is a progress from the complex to the simple, I shall follow a direct physiological sequence, in the development order, and beginning with the lesions of the peripheral nerves, shall proceed to those of the spinal cord, and lastly to those of the eucephalon, passing upward from the simpler to the more complex.

To quote the language of a recent English authority, "the inductions which we get by observing the simple, may be used with success to disentangle the phenomena of the complex; but the endeavor to apply the complex and obscure to the interpretation of the simple, is sure to end in confusion and error." The higher faculties are formed by evolutions from the more simple and elementary, just as the more special and complex structure proceeds from the more simple and general; and in the one case, as in the other, we must, if we would truly learn, follow the order of development.

From the intimate correlation of every portion of the organism, by means of the nervous system, with every other, you will readily understand that it is impossible for any one portion to be diseased without interfering to some extent with the health of all the others. This is a general proposition, applicable to all diseases and derangements whatever. Now when this internuncial agent, by means of which this correlation is maintained, becomes itself the seat of disease primarily, either in its centres or in its peripheral ramifications, the disturbances of all the other portions of the organism will be so much the more certain, so much the more prompt, and so much the more clearly manifested.

Our knowledge of the diseases of the nervous system has followed, step by step, advances which have been made in our acquaintance with its physiology.

But few years, comparatively speaking, have elapsed since the pathology of the nervous system began to be investigated as a distinct department of medical science. Formerly, symptoms which are now traced to their origin in diseases of the nervous system, to quote the quaint expression of Sir Charles Bell, were classed as "*nervous* symptoms," which was another way of saying that the physician was not expected to investigate or explain them.

In using such language in 1811, the father of modern physiology could scarcely have expected its applicability to continue in force for more than half a century; but what was true of all, or nearly all, at that day, is unfortunately true of many to-day, and the epithet, "nervous," has at this time come to be considered an excuse for professional ignorance.

Indeed, the whole subject was a terra incognita previous to the appearance in 1811 of Sir Charles Bell's little work, entitled "An Idea of a New Anatomy of the Brain, submitted to the observation of the author's friends."

Previous to this era, it is true, the way had been prepared by Monro, previous to 1767, who discovered that the ganglia of the spinal nerves were formed on the posterior roots, and that the anterior roots passed by the ganglia. Santormi and Wrisberg observed the two roots of the fifth pair, and Prochaska, in his work, "de Structura Nervorum," in 1779, and Sœmmering, noticed the resemblance between the spinal nerves and the fifth pair. But these discoveries had remained useless and sterile in their hands, having neither been traced backward to any physiological principle of action, nor forward to any function in the animal economy. But to Sir Charles Bell may with justice be conceded the honor of having initiated a new era, the era of accurate observation and logical induction.

Coincidentally with Bell, Magendie, in France, published his experiments on the roots of the spinal nerves and on the fifth pair. (The claim of priority of discovery was disputed by these observers.) Flourens and Longet in France, and Muller in Germany, labored assiduously in the same field, and made valuable contributions to this branch of medical science. But for the next great step in the knowledge of the nervous system, we are indebted to Marshall Hall, whose discovery of the functions of the true spinal or excito-motor division of the nervous system has thrown a flood of light upon the field of pathological investigation, not exceeded in brilliancy by that of Bell.

In our own day, rapid strides are being made daily, almost, in this department of pathology, and the researches of Solly, Reid, Jones, Seiveking, Ratcliffe, Reynolds, Claude Bernard, Rabuteau, Cyon and Ludwig, Meyer, Sequard, and a host of others, have given a greater impulse in this direction than has ever been imparted to it in any one generation. The investigations of Brown Sequard upon the pathology of the spinal cord and sympathetic nerve, and those of Campbell in our own country upon the nature of continued fevers and their nervous relations, and upon the excito-secretory system, have been especially valuable. And still later, Hammond has brought down the data of science to the present day, and given us the gist of all that is known upon the subject.

The ætiology of the diseases of the nervous system is coextensive with that of diseases in general. Without attempting to dogmatize upon this subject, by endorsing the theory of the neuropathists, of the invariable nervous origin of diseases, it is reasonable to say, that, if diseased processes do not invariably commence in disturbances of the nervous system, it is undoubtedly through this organism that they first declare themselves; and hence, for the causes of nervous derangements you may look to all the possible morbific agencies that the Professor of Practical Medicine has enumerated in his comprehensive ætiological series. But remember this, which you may impress upon your minds as an axiom, that for every variation and innervation, for every modification of function, there must be a corresponding modification of structure; perfection of functional energy, which is force in action, essentially involves integrity of structrue. What is true of the mass, is true of its constituent parts.

Just as in cerebral hæmorrhage, when the laceration and destruction of the brain-substance involves total suspension or destruction of sense and motion, of all cerebral action, so also in epilepsy, that yet unsolved enigma, rest assured there is change in cerebral structure, even though it may yet elude the most delicate microscopic investigation. The error is not one of generalization, (the results of logical induction are more accurate than those of microscopic observation), but of observation.

The truth of this proposition is self-evident from such a multitude of observations, that when other observed facts seem to be incomprehensible under the law, it is safe to lay them aside as unclassified facts, with the certainty that with improved means of observation, and broader bases of classification, they will eventually be reducible to the general law.

This proposition, indeed, underlies the phenomena of life itself. Life is not structure, organization, but is conditioned thereupon. Force in action, manifested through organization, is the expression of life.

In your consideration of the innumerable phases of nervous disease which will be presented to you in your future career as practitioners, there will be found, to borrow the language of mathematics, one constant factor. And a knowledge of this will materially aid you in forming your diagnosis. Bear this well in mind, that, no matter what may be the apparent symptoms, whether indicating disturbance of function in the direction of excess or defect, convulsion or paralysis, delirium or coma, the one constant element is debility, depressed vitality, loss of force. There can be no such thing as plus vitality. The maximum result of the co-operation of perfect functional equilibrium, with perfect structural integrity (for indeed the one condition necessarily involves the other), is perfect health; beyond this point it is impossible to go. Hence, there is no such condition as plus vitality. True, one function may be performed in excess of the normal standard, but it will be at the expense of all the others. Muscular motions may be performed excessively, as in the case of convulsions, but it is because the functional energy of the hemispheres is too feeble to maintain their domination and control by means of which the spinal cord is kept in subjection to the will. On the other hand, the hemispheres may be irritated to excessive activity; cerebration may be performed with apparent lightning-like rapidity, as is seen in some phases of delirium and of insanity, but it is at the expense of coherence and intellection.

In the course of these lectures, gentlemen, I shall not detain you with quotations from authorities to sustain my positions. The truth or falsehood of these I shall expect you to demonstrate to your own satisfaction by reference now to the standard authors within your reach. It will be my endeavor to give you simply general outlines, covering as large a space as possible, which you will fill in for yourselves by subsequent study.

A systematic study of nervous pathology, ought to begin, as a first step, with an investigation of the derangements of the sympathetic or ganglionic nervous system, so called. But unfortunately, medical literature is singularly barren upon this subject. Owing to the intimate dependence of organic processes upon the integrity of this division of the nervous system, and the extreme danger to the life of the animal, incurred as a consequence of any experiments upon it, little has been done in the way of the determination of its function by actual experiment; hence, but little progress has been made in comparison with what has been learned in other branches of this department of pathology. Indeed, from the very intricacy of the correlations, it is almost impossible to isolate the pathology of the sympathetic from that of the rest of the organism. What we know of the pathology of this portion of the nervous system—for which we are indebted to Pourfour du Petit, Magendie, Alcock, Claude Bernard and Brown Sequard principally—may be condensed briefly thus: In cases where the influence of this portion of the nervous system is withdrawn by disease or injury, the effects manifested are dilatation of the blood-vessels, afflux of blood, and increase of vital properties. These effects are likewise produced by certain drugs. When, on the contrary, the influence of this nerve is increased by irritation, either mechanical or galvanic, for example, there will ensue contraction of the blood-vessels, diminution of blood, and decrease of vital properties.

This general fact is applicable practically, thus: in those conditions characterized by dilated blood-vessels, various congestion, general and local, we assume the proximate cause to be paralysis of the sympathetic nerve; and hence, the proper remedies would be those which would contract the blood-vessels—hence the great value of the bromides.

The pathology of this portion of the nervous system is best known through the effect of injuries to the fifth pair of nerves, and the pneumogastric.

Injuries to the former frequently result in disorganization of the eyeball, commencing with opacity of the cornea, and ending with total destruction of the organ whose nutrition is dependent on the integrity of the ciliary or ophthalmic ganglion of the sympathetic. This has been demonstrated, experimentally, often.

So too, compression, or section of the cervical ganglia of the sympathetic is followed by contraction of the pupil, retraction of the ball of the eye, and consecutive inflammation.

On the other hand, irritation of the cervical sympathetic, either mechanical or galvanic, will be followed by dilatation of the pupil and an anæmic condition of the cornea.

It is probable, although it has not yet been demonstrated by actual observation, that injury to the ganglia on the posterior roots of the spinal nerves, in infancy, or in intra-uterine life, may be the cause of defective growth and nutrition in children.

The relation of the ganglionic nervous system to the *heart*, is demonstrated very frequently in the course of surgical practice. Experiment has demonstrated, that crushing the brain or spinal cord occasions a sudden cessation of the heart's action. But the same effect is produced by crushing the stomach, or a limb, or the foot; and this, too, after the careful removal of the brain and spinal cord; from which it is clear that the effect could have been produced by the influence of the sympathetic alone. Some of the cases of sudden death occurring after surgical operations, or after injuries by no means essentially mortal, can be explained in no other way.

I can recall at this moment one such instance within my own observation in which death resulted from the passage of a wagon wheel over the knee of the patient, crushing the joint. This is what is termed *shock*, and it is exemplified sometimes under the influence of intense mental excitement, particularly where the heart is already weakened by pre-existing disease.

In pursuance of the plan indicated to you already, i. e., to begin with the simplest form of derangement of innervation, and proceed gradually, in the order of physiological development, to the more complex, we will begin our studies with those disturbances of innervation involving primarily and essentially the sensory fibres of the peripheral nerves, and the function of common sensation.

Sensation may be disturbed in three directions: by diminution or abolition, by exaltation, and by perversion.

The disturbance of innervation by diminution of its intensity, or its total abolition, is that condition known to pathologists as *Anæsthesia*, a word derived, like most of our scientific technology, from the Greek, meaning loss of feeling. We frequently find this condition expressed by the term "paralysis of sensation," but much confusion will be avoided by the abolition of the term paralysis from this association, and by limiting its application to loss of motor power alone.

Of the various forms of anæsthesia, we will first consider peripheral, or local anæsthesia; and this may be occasioned by any one of three conditions:

*First.* Morbid changes in the extremities of the nerve or nerves implicated, by means of which they are incapacitated from receiving normal impressions, as illustrated by the numbness produced by intense heat or cold, or by the action of certain chemical agents, such as carbolic acid, applied to the surface.

*Second.* By incapacity in the sensory or afferent fibres of a nerve to convey normal impressions to the brain, as illustrated by the interception of this conducting power by means of a ligature surrounding a limb, or by section, or by crushing or bruising of a nerve. And—

*Third.* The third condition which would be manifested by anæsthesia, must be change in the nerve cells of the central ganglia, so that, when an impression is received at the extremity of a sensory or afferent nerve, and conveyed to the centre, it is not received there by reason of some modification in the structure of the nerve cells at that point. This condition will be considered subsequently, when we come to consider diseases of the nervous centres, to which category it more properly belongs.

Anæsthesia may be localized topographically, according to the site of its manifestations, into—

1st, Cutaneous; 2d, Muscular; 3d, of the Fifth Nerve; 4th, Sensorial; and 5th, Visceral Anæsthesia.

The first, and by far the most common form, is that in which the skin is involved, and is manifested in deficiency or total loss of tactile sensibility and of perception of temperature; under which the subject cannot distinguish hot bodies from cold. Under this condition much injury to the cutaneous surface may be received by contact with very hot or very cold bodies.

It sometimes results as a consequence of pressure, as illustrated in the familiar instance of bedsores, although in this condition something further is involved, i. e., modifications of the nutritive nerves.

Anæsthesia of the fifth pair of nerves, is dependent on lesions of that nerve at points in its course which are indicated by the extent of the abolition of sensation.

Thus, if sensation be lost over an extent of surface dependent for its nervous supply upon more than one division of the nerve, the lesion must be located at a point in its course posterior to its division into its separate branches. If, again, the loss of sensation be accompanied by loss of motion in the muscular apparatus supplied by the fifth pair, the lesion must be located at the ganglion of Gasser, or at some point in the course of the nerve posterior thereto.

Or, to go still farther, if the anæsthesia be associated with disturbance of the functions of other nerves contiguous thereto, the seat of the disease must be in the brain itself; in which case, the importance of the symptom becomes more serious, for which Romberg is our authority. This condition sometimes precedes or succeeds its opposite condition, hyperæsthesia, neuralgia; and it is sometimes also associated with paralysis.

That form of local anæsthesia which we have termed *sensorial* anæsthesia—manifested in the nerves of special sensation, the olfactory, the optic, the auditory, the gustatory—belongs more properly to special branches of pathology, and we shall be obliged to omit its consideration.

Anæsthesia of the mucous surface and of the viscera, is probably dependent on lesion in the sympathetic nerve, of which so little is known that I can give you no positive instructions upon the subject. As a local disease, this condition is neither very common nor very important, but understanding its elementary and simpler forms, you will be better able to appreciate its value when it occurs as a symptom, as a factor, in more complicated conditions.

Let us fix thoroughly and firmly in our minds the definitions of simple morbid conditions, the letters and syllables of disease, and we shall be able to spell the "big words" easily when they come before us.

The diagnosis of anæsthesia must not be made up exclusively from the statements of patients; instrumental intervention will be found necessary frequently. And for this purpose, various instruments have been proposed by different authorities, all, however, consisting of modifications of the same radical idea, i. e., two sharp points, which are to be brought into contact with the skin at the same moment, that their distance from each other may be estimated by the patient, and this estimate will constitute the index of cutaneous sensibility.

These instruments are termed æsthesiometers. That of Dr. Sieveking, commended by most writers, is simply a beam compass; that is, a graduated metal bar, four or five inches long, upon one end of which is fixed at right angles to the axis of the bar, a steel point, while a slide bearing another steel point traverses the bar. That of Dr. Hammond consists of an ordinary pair of dividers, having a graduated arc affixed to one arm. I have very frequently made use of the separated points of a pair of scissors or two toothpicks, to acquire the same information.

There is one point of practical importance to which I must allude, with which it is desirable that you should familiarize yourselves by actual experiment. I mean the normal difference in the sensitiveness of different portions of the surface of the body.

The distances at which the points of the æsthesiometer can be distinguished, vary from half a line at the point of the tongue to thirty lines at the middle of the arm, thigh, back and neck.

The treatment of local anæsthesia depends of course upon the cause; this being removed, the malady will generally subside. As in the case where it depends upon spasm of the blood-vessels, anæmia, etc., those agents which have been found to improve nutrition, will be found most beneficial—strychnia, mineral tonics, quinine, etc.

Some of the more recent writers upon this subject, Meyer, Hammond, Ratcliffe and others, place more reliance upon electricity than upon any other remedy, and this in the form of the primary current. It is certain that this agent is destined to play a very active part in the neuro-therapeusis of the future.

